# Factors Associated with Late Antenatal Initiation among Women in Malawi

**DOI:** 10.3390/ijerph21020143

**Published:** 2024-01-27

**Authors:** Martin Enock Palamuleni

**Affiliations:** Population and Health Research Entity, North-West University, Mafikeng 2735, South Africa; martin.palamuleni@nwu.ac.za

**Keywords:** antenatal care (ANC), late ANC initiation, logistic regression, Malawi

## Abstract

**Background** Early initiation of antenatal care (ANC) is critical in identifying and mitigating adverse pregnancy-related complications. However, globally, a high percentage of women initiate ANC only at a late stage of their pregnancy. In view of this, the main objective of the study is to establish the prevalence and factors associated with late ANC initiation among women in Malawi. **Methods** The study was based on the 2015–16 Malawi Demographic and Health Survey (MDHS). The study population consisted of 13,251 women of reproductive age who had given birth during the five years preceding the survey. The data was analyzed using the chi-square test and multivariate logistic regression. **Results** The prevalence of late ANC initiation in Malawi was 75.6%. The logistic regression modelling revealed increased odds of late ANC initiation attendance among women residing in the Northern Region (AOR: 1.172; 95% CI: 1.021–1.345) and the Central Region (AOR: 1.178; 95% CI: 1.074–1.291), women residing in urban areas (AOR: 1.273; 95% CI: 1.108–1.463), women with no education (AOR: 1.814; 95% CI: 1.13–1.47) or with primary education (AOR: 1.697; 95% CI: 1.13–1.47), women with less than four ANC visits (AOR: 4.155; 95% CI: 4.002–4.814), unmarried women (AOR: 1.478; 95% CI: 1.111–1.985) and those whose last birth was not by caesarean section (AOR: 1.377; 95% CI: 1.179–1.607). Reduced odds of late ANC initiation among women were observed among women in the 20–24 age group (AOR: 0.634; 95% CI: 0.456–0.881), those in the 25–29 age group (AOR: 0.645; 95% CI: 0.476–0.874) and those aged 30–34 years (AOR: 0.634; 95% CI: 0.456–0.881). **Conclusions** The study found that ANC initiation in Malawi is often delayed, with most first visits occurring after the first trimester. Late ANC initiation is associated with region, place of residence, marital status, and the women’s age. These are significant factors to be considered when designing new or reviewing ANC policies and strategies aimed at increasing ANC utilization and encouraging early initiation of ANC. Earlier ANC initiation among Malawian women can contribute positively towards improving maternal and child health in Malawi. Therefore, government policies and interventions should target women with no or little education, those living in poor families and other modifiable risk factors, such as young unmarried women.

## 1. Introduction

Despite noticeable improvements in maternal and child health in Malawi, the estimates of maternal and child morbidity and mortality remain high by global standards. There are researchers who regard the indicators to be amongst the worst in the world [1]. The Maternal Mortality Ratio (MMR) increased from 620/100,000 in 1992 to 1120/100,000 in 2000, but it declined to 984/100,000 in 2004, 675/100,000 in 2010 and 439/100,000 in 2015 [2,3,4,5,6]. At the same time, the mortality rates of children aged under five declined from 234/1000 in 1992 to 203/1000 in 2000, 133/1000 in 2004, 112/1000 in 2010 and 63/1000 in 2015 [2,3,4,5,6]. The World Health Organization (WHO) estimated that in 2019, the MMR in Malawi was 381/100,000 [7].

The estimates presented in the preceding paragraph indicate the continued challenging maternal and child health indicators in Malawi. These can be ascribed partly to the limited use of maternal and child health services. The percentage of pregnant women who had at least four antenatal visits was 63.8% in 1992, 56.7% in 2000, 57.7% in 2004, 44.5% in 2010 and 50.6% in 2016, whereas assistance during delivery by a skilled attendant increased from 53.9% in 1992 to 55.8% in 2000, 75% in 2010 and 90% in 2015 [8]. Although antenatal care (ANC) utilization has increased again over the past years in Malawi, the MMR remains high. These estimates suggest the need to continue encouraging pregnant women to make use of antenatal services, as well as of maternal and child health services.

One of the strategies used to ensure the continued decline of MMR is by increasing the use of ANC. Earlier studies have revealed that ANC services are the most cost-effective intervention for reducing the MMR in developing countries [9,10]. ANC is regarded as a preventive healthcare measure in that prescribed routine medical check-ups are provided during pregnancy by a skilled attendant to timely detect and address any problems.

Three aspects of ANC are regarded as critical for maternal and child health services. These are the number of ANC visits, the availability of ANC by a skilled professional and the place of ANC services. Studies on the number of ANC visits have focused on the timing of first antenatal visits [11], adherence to the recommended minimum number of visits [8,12,13] and the quality of care. This study focused on the timing of the first antenatal visit. For this study, early initiation is regarded as a visit during the first trimester of the pregnancy, while late initiation is regarded as ANC visits initiated after the first trimester. Early initiation of ANC means the timely provision of maternal health information and services according to the gestational age and health condition, including iron supplementation, deworming tablets, tetanus injections and malaria prophylaxis (4). On the other hand, late ANC initiation means that pregnant women miss the opportunity to receive health information and interventions, such as the early detection of HIV, malaria and anemia prophylaxis, and prevention or management of complications.

Late initiation of ANC is not only associated with negative health outcomes such as premature birth, still birth, low birth weight and increased complications during pregnancy and childbirth, but it also leads to the poor use of other services such as delivering in health facilities and using skilled attendants during delivery [14]. The World Health Organization (WHO) revised the recommended minimum number of ANC visits from four to eight, with the first visit to take place in the first trimester [15,16].

Although studies have been conducted on the prevalence and determinants of maternal health services in Malawi [8,14,15,16], few studies investigated late ANC initiation, and no study has used the Demographic and Health Survey (DHS) datasets. The few studies that have been conducted are qualitative, hospital or district based, involving small sample sizes [17,18]. There is a paucity of studies using large-scale datasets. This study used a large-scale national and representative data from the 2015–16 Malawi Demographic and Health Survey (MDHS) to examine the correlates of late ANC initiation among Malawian women in the reproductive age group. Planners and policymakers can use the results of this study to design programs for Malawi that contribute towards the successful adoption of earlier first ANC visits.

### Conceptual Framework

**Figure 1:**
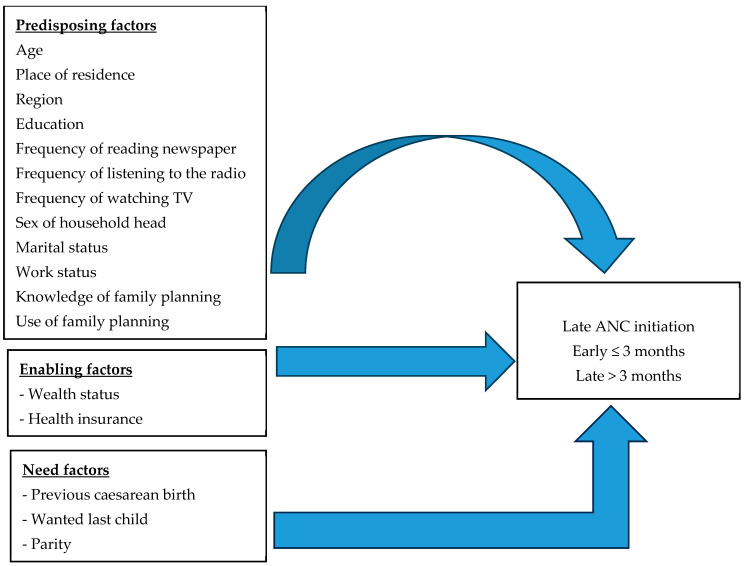
Modified conceptual framework for late ANC initiation.

Andersen’s behavioural model for the utilization of health services was used to study the determinants of late ANC initiation among women in Malawi [19,20,21]. Andersen’s framework suggests that late ANC initiation is a function of predisposing, enabling and need factors (see Figure 1). The predisposing factors include the background characteristics of individuals that exist before the need for the utilization of antenatal care. Studies have shown that factors are age [22,23], education [24,25], region [22], marital status [23,26], ethnicity [26,27], place of residence, parity [23], unplanned pregnancy [28], wanted last child [29] and previous history of caesarean section delivery [30]. The enabling factors refer to the logistical aspects of obtaining ANC. It has been reported that timing of ANC is significantly associated with wealth status [24] and being covered by medical insurance [31]. Women from rich households are more likely to initiate ANC early than women from poor households [24,27,28,32,33,34]. This is the case because women living in wealthy households can afford the cost of medical care. The need factors are those that threaten the lives of mothers and their babies, and these affect the use of early ANC. Studies show that if the pregnancy was unintended or the child was not wanted, then the chances were higher for late initiation of ANC [35,36]. Women whose previous birth was by caesarean section tended to initiate ANC early [28,37,38], whereas women who had given birth to a large number of children tended to initiate ANC late [39].

## 2. Methods

### 2.1. Study Setting

Malawi is a small country in southeast Africa. Its land area is 118,484 square kilometres and comprises 3 administrative regions (Northern Region, Central Region and Southern Region) and 28 districts. The population of Malawi increased from 13,077,160 in 2008 to 17,563,749 in 2018, implying a growth rate of 2.9% per year. The demographic indicators are among the worst in the world.

### 2.2. Data Source

The study used data extracted from the 2015–16 MDHS. The 2015–16 MDHS was carried out by the Malawian National Statistical Office with financial and technical assistance from ICF Macro [6].

The data were collected from eligible women aged 15–49 years who were residents in the respective household 24 h prior to the survey. The data collection made use of a two-stage stratified cluster sampling technique [6]. The first stage selected 850 enumeration areas (EAs), comprising 173 EAs in urban areas and 677 EAs in rural areas, using probability proportional to the size of the areas. The EAs were based on the 2011 Malawi Population and Housing Census [6]. The second stage of the data collection phase involved selecting 30 households per urban cluster and 33 per rural cluster, with an equal probability of systematic selection from the household listing. All women in the reproductive age group were eligible to be interviewed, irrespective of whether they were visitors or permanent residents. The survey identified 25,146 eligible women of reproductive age, and a total of 24,562 women were interviewed, achieving a successful response rate of 97.7%. A detailed report of the sampling procedure followed can be obtained from the 2015–16 MDHS report [6].

### 2.3. Study Population

The study population consisted of 13,251 women aged 15–49 years who had given birth in the five years preceding the survey. All records with missing cases were excluded from the analysis.

### 2.4. Variables

#### 2.4.1. Dependent Variable

The dependent variable for this study was the late initiation of ANC, when the first ANC visit occurred only in the second or third trimester. To establish the variable, the interviewed women were asked the following: “How many months pregnant were you when you first received ANC for this pregnancy”? The response to this question ranged from 0 to 10 months. Based on the WHO’s recommendation to initiate the first ANC visit in the first trimester, this variable was classified and coded into two groups: an early initiation of ANC being the first visit having taken place during 0 to 3 months, while late initiation of ANC being the first visit during 4 to 10 months.

#### 2.4.2. Independent Variables

The independent variables were identified from previous studies [12,25,38,40,41,42]. The following independent variables were included in the analyses: the participant’s age, region, place of residence, education, sex of head of household, wealth status, work status, marital status, parity, frequency of listening to the radio or watching television or reading newspapers/magazines, knowledge of family planning, ever use of family planning, wanted last child and last birth by caesarean section. These independent variables were categorized as follows: the participant’s age was recorded in five standard year groups (1 = 15–19, 2 = 20–24, 3 = 25–29, 4 = 30–34, 5 = 35–39, 6 = 40–44, 7 = 45–49); region (1 = Northern Region, 2 = Central Region, 3+ Southern Region); place of residence (1 = urban, 2 = rural); education (1 = no education, 2 = primary, 3 = secondary, 4 = higher); sex of head of household (1 = male, 2 = female); wealth status (1 = poorest, 2 = poorer, 3 = middle class, 4 = richer, 5 = richest); work status (1 = not working, 2 = working); marital status (1 = never married, 2 = married, 3 = formerly married); parity (1 = no children, 2 = 1–2 children, 3 = 3–4 children, 4 = 5 children and more); frequency of listening to the radio (1 = not at all, 2 = less than once a week, 3 = at least once a week); frequency of watching television (1 = not at all, 2 = less than once a week, 3 = at least once a week); frequency of reading newspapers/magazines (1 = not at all, 2 = less than once a week, 3 = at least once a week); wanted last child (1 = wanted then, 2 = wanted later, 3 = wanted no more); last birth by caesarean section (1 = no, 2 = yes); knowledge of family planning (1 = knows no family planning, 2 = knows family planning); ever use of family planning (1 = never used family planning, 2 = ever used family planning).

### 2.5. Analysis

Three statistical methods were used to analyze the data. First, frequency distributions were determined for all the variables to scrutinize the background features of the study population. Second, cross tabulations and the chi-square (χ2) test were performed to examine the relationships between late ANC initiation and the independent variables. Third, multiple logistic regression was used to assess the effect of variables on the late ANC initiation while controlling for potential confounding factors. The data were analyzed using the Statistical Package for Social Sciences (SPSS) version 22.0.

We also checked the presence (or absence) of multicollinearity among the independent variables by using the variance inflation factor (VIF), adopting the cut off value of 2. All the independent variables had a VIF of less than 2, implying that there was no multicollinearity between the variables. The mean VIF was 1.14.

### 2.6. Ethical Consideration

This study is an analysis of secondary data collected during the 2015–16 MDHS. Therefore, the authors did not need to apply any ethical considerations regarding the study’s participants. However, the organization of the 2015–16 MDHS (including sampling methods, instruments and data collection) involved ethical approval from the Institutional Review Board of ORC Macro Inc. and the National Health Sciences Research Committee in Malawi [6]. Before those interviews were conducted, informed consent from the participants was obtained and the participants were assured of strict anonymity and confidentiality. They had the autonomy to leave the study at any time. For the purposes of this study, the author sought permission to use the findings from the 2015–16 MDHS from Measure DHS, which was granted.

## 3. Results

### 3.1. Demographic Characteristics of the Participants

**Figure 2:**
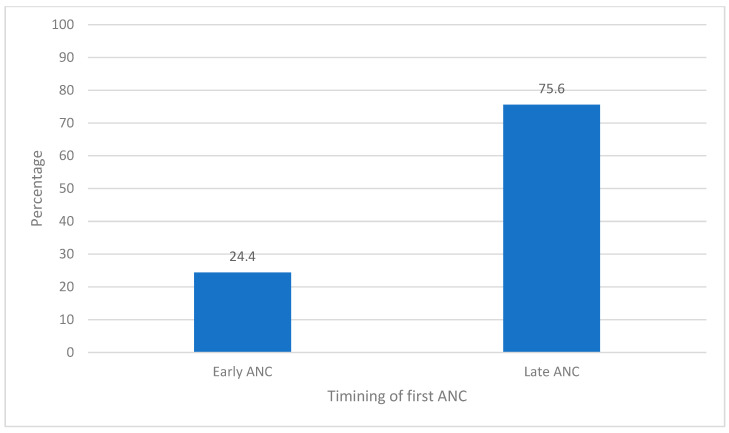
Timing of first visit for antenatal care (ANC), Malawi 2015–16.

**Figure 3:**
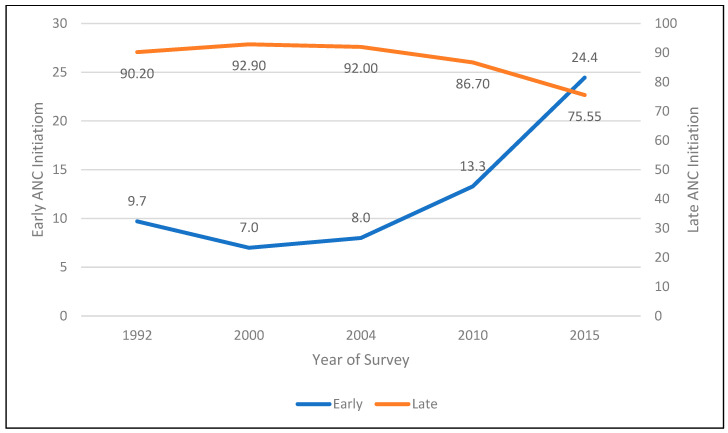
Trend in timing of first antenatal care (ANC) visit, 1992–2015.

The study population consisted of 13,251 women of reproductive age. The percentage distribution of the study population increased from 8.6% in the age group 15–19 years to a local maximum of 28.6% in the age group 20–24 years and declined steadily reaching 2.2% in the age group 45–49 years. The mean age of the population was 28 (±7) years. Most of the women were married (82.9%), working (66.3%), only attained primary education (65.4%) and lived in rural areas (85.5%). Most of the study population never read newspapers (82.4%), did not listen to the radio (52.3%) and did not watch TV (82.3%), while 45% were considered poor. Nearly all the interviewed women knew about methods of family planning (99.8%), although only 86.3% of them reported ever using family planning methods. Slightly over half of the sample (51.6%) adhered to four or more ANC visits, and about three-quarters (75.6%) of the women-initiated ANC after the first trimester (Figure 2). Similar percentages from earlier DHSs are 90.2% in 1992, 92.9% in 2000, 92.0% in 2004 and 87.6% in 2010, respectively. These statistics indicate that the percentage initiating ANC late seemed to be declining since 2004 (Figure 3).

### 3.2. Association of Late Uptake of ANC and Participants’ Characteristics

Table 1 also shows the findings of the bivariate analysis comparing the association of certain factors with late initiation of ANC. The variables that were significantly associated with late ANC initiation are age, education, frequency of listening to the radio, frequency of watching television, marital status, ever use of family planning, adequate ANC visits, sex of household head, wealth status, parity, wanted last child and last child by caesarean section.

As age increases, the percentage initiating ANC late declines, reaching the lowest level in the 30–34 age group and increasing thereafter. The results indicate that late ANC initiation is negatively correlated to education level and wealth status. The percentage of women initiating late ANC is highest in the Central Region (76.3%) and lowest in the Southern Region. More women in the rural areas initiate ANC late than women living in urban areas. Unmarried women initiate ANC later than married women. The percentage of women initiating ANC later is highest among women who have never used contraceptives (77.8%), women whose last birth was not by caesarean section (76.2%) and women who wanted their last child later (78.7%).

### 3.3. Determinants of Late ANC Initiation in Malawi

The relationship between late ANC initiation and the background variables was further explored by using logistic regression modelling. Table 2 indicates that late ANC initiation is significantly associated with maternal age, region, type of residence, the mother’s education level, the number of visits, wanted last child, last child by caesarean section and marital status.

The results indicate that women in the 20–24 age group were 37% less likely to initiate ANC late compared with women in the 45–49 age group (AOR: 0.634; 95% CI: 0.456–0.881). Women in the 25–29 age group were 35% less likely to initiate ANC late compared with women in the 45–49 age group (AOR: 0.645; 95% CI: 0.476–0.874). Women in the 30–34 age group were 36% less likely to initiate ANC late compared with women in the 45–49 age group. The results show that the odds ratios decreased with the increasing age of the woman up to the 30–34 age group and then increased as the age of the women increased. Women residing in the Northern Region (AOR: 1.172; 95% CI: 1.021–1.345) and the Central Region had 17% increased odds of initiating ANC late compared with women residing in the Southern Region (AOR: 1.178; 95% CI: 1.074–1.291). Women residing in urban areas had 27% increased odds of initiating ANC late compared with women residing in rural areas (AOR: 1.273; 95% CI: 1.108–1.463). Women with no education had 81% increased odds of late ANC initiation (AOR: 1.814; 95% CI: 1.13–1.47) compared with women with higher education. Women with primary education had 70% increased odds of late ANC initiation (AOR: 1.697; 95% CI: 1.13–1.47) compared with women with higher education. Women with only secondary education had 79% increased odds of late ANC initiation (AOR: 1.793; 95% CI: 1.390–2.312) compared with women with higher education. Women with less than four ANC visits had 16% significantly increased odds of late ANC initiation (AOR: 4.155; 95% CI: 4.002–4.814). Unmarried women had 48% significantly increased odds of late ANC initiation (AOR: 1.478; 95% CI: 1.111–1.985) compared with women who were formerly married. Women whose last delivery/birth was not by caesarean section had 38% increased odds of late ANC initiation (AOR: 1.377; 95% CI: 1.179–1.607). 

## 4. Discussion

The main finding of this study was the persistently late ANC initiation in Malawi. The data analysis revealed that the prevalence of late ANC initiation is 75.5%. Similar percentages from earlier MDHSs are 90.2% in 1992, 92.9% in 2000, 92.0% in 2004 and 87.6% in 2010 [2,3,4,5]. Figure 3 indicates that even though late initiation of ANC is declining, the percentage of women initiating ANC late is still very high.

The high prevalence of late initiation of ANC in Malawi is comparable with what is found in other sub-Saharan countries, namely: 72.7% in the Democratic Republic of Congo (2015), 77.6% in Gambia (2013), 78.1% in Liberia (2013), 73.8% in Zambia (2013–2014), 73.5% in Tanzania (2015) and 78% in Nigeria (2013). The incidence was found to be lower in Angola (41.4%) and Zimbabwe (55.0%). Adherence to traditional culture may be a hindrance to women using ANC services, including their first ANC visit within the first trimester. In most African societies, culture often prevents women from discussing pregnancy matters with other people for fear ‘that something may be done to harm the mother and unborn child’ [43,44,45,46]. In addition, African culture dictates that women may discuss pregnancy and be willing to initiate ANC only once the pregnancy is visible [47].

The study found that age of the participants was significantly associated with late initiation of ANC. Older women tend to be more likely to initiate ANC late as compared with younger women. The importance of the participants’ age was also underscored in other studies [24,25,38,42,48,49]. One plausible explanation is that young women may be better educated than older women, and, as such, they may have acquired more knowledge on the need to initiate ANC within the first semester. Another explanation could be that older women may initiate ANC late because of the confidence acquired from their experience of previous childbirth or pregnancy [29]. Third, older women may be assumed to have more children and may have more time and resource challenges to visit health facilities.

Region of residence is significantly associated with late initiation of ANC in Malawi. Late initiation of ANC is highest in the Central Region, followed by the Northern Region and lowest in the Southern Region. Other studies conducted in Malawi have reported regional differences in the utilization of maternal health services. In addition, studies conducted in Burkina Faso [50], Ethiopia [11,42], Ghana [22], Nigeria [27,51] and Sierra Leone [52] have reported regional differences in the initiation of ANC. Two factors could be responsible for the observed regional differences in the late initiation of ANC. First, the availability and accessibility of maternal healthcare facilities could differ between regions. Previous studies have shown that distance to the healthcare facility may deter or encourage women to initiate early ANC. It is expected that late initiation of ANC could be common in regions where antenatal clinics are not accessible. Second, the characteristics of the regional populations could also be responsible for the observed regional disparities. Regions recording a high number of illiterate and uneducated populations could be expected to have a high percentage of late ANC initiators. In Malawi, late initiation of ANC would be expected to be highest in the Southern Region, followed by the Central Region and lowest in the Northern Region. The fact that this is not the case may be an indication that there are other factors, such as traditional culture and availability/accessibility of healthcare facilities, influencing late initiation of ANC.

Another important determinant of late initiation of ANC in Malawi tends to be education. Women with low levels of education are more likely to initiate ANC late than women with higher levels of education. These findings are comparable to those reported by other researchers [11,24,25,27,28,32,33,34,36,48,50,51,53]. Three reasons may be suggested as explanations for why uneducated women are likely to initiate ANC late. First, educated women are aware of the merits of early initiation of ANC. Second, educated women not only attach greater value to their health, but they also tend to have greater decision-making powers on health-related matters and thus also the early initiation of ANC. Third, although the head of the family or household is expected to decide on the use of ANC, especially in rural and traditional African societies, educated mothers may have the confidence and courage to negotiate with the family or the household to use ANC. Fourth, better educated women are known to make independent decisions regarding both when and where to use ANC services.

Women who made fewer than four ANC visits were more likely to initiate ANC later than those with four ANC visits or more. Similar findings were reported in other studies [41,54,55]. Women with fewer than four ANC visits may have experienced challenges to access maternal health services or had negative healthcare-seeking behaviour in terms of modern healthcare services. It can be argued that women making fewer than four ANC visits have insufficient knowledge of ANC. A better informed level of knowledge of ANC has been found to be associated with early initiation of ANC [56,57].

Late ANC initiation was also significantly associated with marital status. This study showed that married women initiate ANC earlier than unmarried women. The finding that unmarried women initiate ANC late has also been found in other studies [23,26,40,47,58]. Late ANC initiation among unmarried women may be due to insufficient resources in terms of transportation to the clinic or any other resources needed to access ANC. The absence of male partners may worsen the situation. Although ANC services are free in Malawi, women still need financial support to access them, especially if they are living in remote areas. The absence of support from a partner may be a hindrance to early ANC initiation. Studies conducted in other countries have reported inadequate resources as a hindrance to early ANC initiation [45,57,59]. There is also a stigma associated with unmarried women who become pregnant, and thus they may hide their pregnancy as long as possible.

The study found that women whose last live birth was by caesarean section are likely to initiate ANC early. This finding is consistent with results from other studies [28,37,38]. One possible explanation for this may be attributable to the fact that decisions to use ANC and its timing are a function of women’s past experience, including complications during pregnancy [47]. On the one hand, women may initiate ANC early if they perceive the likelihood of complications in the pregnancy to be high. On the other hand, if the woman had bad experiences during previous ANC visits, then this may deter her from using ANC in the future or it may result in initiating ANC late. Women who received bad treatment at the clinic during their previous pregnancy usually initiate ANC late [59,60].

The study found that rural women are more likely to initiate ANC early than urban women. A similar observation was made in an earlier study in Malawi [61]. This is unexpected and contrary to findings from other studies [27,38,42,48,49,51,62] as urban women are expected to initiate ANC earlier than their rural counterparts. This stems from increased availability and accessibility of healthcare services and conducive characteristics of urban dwellers. The findings in this study of increased odds of late initiation of ANC in urban areas may arise from the fact that, in Malawi, the level of urbanization is still low and the differences between rural and urban dwellers in terms of social and economic characteristics are virtually non-existent.

## 5. Strength and Weaknesses of the Study

The strength of the current secondary data study is that it is based on a large-scale and nationally representative sample. The study is the first of its kind in Malawi to use DHS data to examine the correlates of late initiation of ANC.

However, the study’s weaknesses need to be acknowledged. First, the study did not collect data on the availability and accessibility of maternal healthcare services. In addition, no data was collected on the reasons for women initiating ANC services late in their pregnancy. Therefore, it is difficult to assess the effect of the individual factors and their interactions. Second, the cross-sectional nature of the dataset used for the study limits the discussions to factors associated with late initiation of ANC and not necessarily the causes thereof. Third, the variables used in this study are affected by recall bias in that the events took place in the past. However, this was minimized by including events occurring during the five years preceding the survey.

## 6. Implications for Policy and Clinical Practice

The study findings are expected to contribute towards policy formulation and implementation regarding maternal healthcare services in general and antenatal care utilization. Policymakers are assumed to formulate policies that will address the issues related to late ANC initiation. This may be done by strengthening the Information, Education and Communication (IEC) program to focus on the advantages and disadvantages of early initiation of ANC.

Future programs should target young unmarried women, especially those residing in the Central Region and the Northern Region. Healthcare service providers, especially those offering ANC services, should be trained to target women in the underserved categories. National health policies should advocate the uptake of ANC services and empower and support healthcare services providers in their important task of promoting maternal healthcare services. The media and organizations working in Malawi to uplift the status of women should be encouraged to raise the awareness on the dangers of late initiation of ANC.

## 7. Conclusions

Following the WHO’s recommendation that pregnant women should initiate ANC within the first trimester, governments in developing countries have initiated programs to encourage early initiation of ANC. However, despite efforts of the Malawian Government, ANC visits are still initiated late in the pregnancy by many women. The late initiation of ANC in Malawi is significantly associated with the age of the participant, education, region, place of residence, marital status and last birth by caesarean section. Based on these findings, the study recommends that the significant factors be considered when drafting new policies or reviewing programs and strategies aimed at promoting early initiation of ANC. Finally, additional research is required to establish the causes and consequences on maternal health services.

## Figures and Tables

**Table 1:** Distribution of the study population by their background characteristics and timing of the first antenatal care (ANC) visit, Malawi 2015–16.

	%	N	First ANC Visit		X^2^	*p*-Value
			Early	Late		
Age (years)					11.80	0.066
15–19	8.6	1134	21.3	78.7		
20–24	28.9	3824	24.8	75.2		
25–29	23.7	3143	24.8	75.2		
30–34	19.7	2606	25.9	74.1		
35–39	12.1	1600	23.9	76.1		
40–44	5.2	683	22.5	77.5		
45–49	2.0	262	22.1	77.9		
Region					3.29	0.193
Northern Region	11.8	1565	24.3	75.7		
Central Region	42.6	5642	23.7	76.3		
Southern Region	45.6	6043	25.2	74.8		
Place of residence					2.05	0.153
Urban	14.5	1919	25.7	74.3		
Rural	85.5	11,331	24.2	75.8		
Education					62.01	0.000
No education	12.4	1637	21.9	78.1		
Primary	65.4	8669	24.2	75.8		
Secondary	20.2	2682	24.8	75.2		
Higher	2.0	262	44.3	55.7		
Frequency of reading newspaper					4.64	0.098
Not at all	82.4	10,922	24.1	75.9		
Less than once a week	10.9	1438	25.5	74.5		
At least once a week	6.7	890	27.0	73.0		
Frequency of listening to radio					8.13	0.017
Not at all	52.3	6930	23.7	76.3		
Less than once a week	18.3	2430	24.1	75.9		
At least once a week	29.4	3891	26.1	73.9		
Frequency of watching TV					17.97	0.000
Not at all	83.8	11,098	23.9	76.1		
Less than once a week	7.4	977	25.1	74.9		
At least once a week	8.9	1176	29.4	70.6		
Sex of head of household						
Male	74.8	9907	24.9	75.1	4.14	0.042
Female	25.2	3344	23.1	76.9		
Marital status					17.89	0.000
Never married	3.8	510	16.9	83.1		
Married	82.9	10,986	24.9	75.1		
Formerly married	13.2	1755	23.6	76.4		
Work status					0.14	0.708
No	33.7	4461	24.3	75.7		
Yes	66.3	8790	24.6	75.4		
Knowledge of family planning					0.44	0.509
No	0.2	31	19.4	80.6		
Yes	99.8	13,220	24.5	75.5		
Ever use family planning					5.80	0.016
No	13.7	1820	22.2	77.8		
Yes	86.3	11,431	24.8	75.2		
Visits					1130.64	0.000
<4	48.4	6418	11.5	88.5		
≥4	51.6	6832	36.6	63.4		
Wealth					24.40	0.000
Poorest	23.3	3094	22.2	77.8		
Poorer	21.7	2879	24.5	75.5		
Middle	19.1	2532	23.1	76.9		
Richer	18.1	2402	26.0	74.0		
Richest	17.7	2344	27.3	72.7		
Last child by caesarean section					41.95	0.000
No	93.5	12,353	23.8	76.2		
Yes	6.5	852	33.7	66.3		
Parity					5.95	0.051
1–2	44.8	5942	25.4	74.6		
3–4	30.1	3987	23.4	76.6		
5+	25.1	3322	24.0	76.0		
Wanted last child						
Wanted then	56.9	7541	26.4	73.6	39.50	0.000
Wanted later	31.1	4117	21.3	78.7		
Wanted no more	12.0	1594	23.4	76.6		
	100.0	13,252	24.4	75.6		

**Table 2:** Factors affecting late initiation of antenatal care (ANC) service utilization, Malawi 2015–16.

Age (years)	Odds Ratio	95% CI	
15–19	0.701	0.487	1.009
20–24	0.634 **	0.456	0.881
25–29	0.645 **	0.476	0.874
30–34	0.636 **	0.479	0.845
35–39	0.707 *	0.534	0.935
40–44	0.778	0.571	1.062
45–49 (Reference)			
Region			
Northern Region	1.158 *	1.009	1.329
Central Region	1.165 **	1.063	1.276
Southern Region (Reference)			
Place of residence			
Urban	1.203 *	1.036	1.396
Rural (Reference)			
Education			
No education	1.814 ***	1.354	2.429
Primary	1.697 ***	1.301	2.214
Secondary	1.793 ***	1.390	2.312
Higher (Reference)			
Frequency of reading newspaper			
Not at all	0.987	0.832	1.172
Less than once a week	1.003	0.820	1.225
At least once a week (Reference)			
Frequency of listening to radio			
Not at all	1.023	0.922	1.136
Less than once a week	1.049	0.925	1.190
At least once a week (Reference)			
Frequency of watching TV			
Not at all	1.029	0.864	1.226
Less than once a week	1.050	0.850	1.296
At least once a week (Reference)			
Sex of head of household			
Male	0.923	0.821	1.039
Female (Reference)			
Marital status			
Never married	1.436 *	1.082	1.904
Married	1.001	0.865	1.157
Formerly married (Reference)			
Work status			
No	0.940	0.857	1.030
Yes (Reference)			
Visits			
<4	4.382 ***	3.995	4.806
≥4 (Reference)			
Wealth			
Poorest	1.117	0.929	1.342
Poorer	0.997	0.835	1.191
Middle	1.082	0.906	1.291
Richer	0.919	0.778	1.086
Richest (Reference)			
Last child by caesarean section			
No	1.377 ***	1.179	1.607
Yes (Reference)			
Parity			
1–2	0.962	0.803	1.152
3–4	1.089	0.946	1.254
5+ (Reference)			
Wanted last child			
Wanted then	0.971	0.839	1.123
Wanted later	1.156	0.988	1.352
Wanted no more (Reference)			

* = *p* ≤ 0.05; ** = *p* ≤ 0.01; *** = *p* ≤ 0.001.

## Data Availability

The dataset, which contains data from the DHS, is freely available to qualified researchers. For this study, a written request was made to the DHS MACRO to acquire the DHS Measure data, and authorization was given to utilize the data. Please apply at https://dhsprogram.com/data/dataset_ad-min/loginmain.cfm (accessed on 12 December 2022) to seek access to the dataset.

## Abbreviations

ANC	Antenatal care
AOR	Adjusted odds ratios
CI	Confidence interval
DHS	Demographic and Health Survey
HIV	Human immunodeficiency virus
ICF	Inner City Fund
IEC	Information, Education and Communication
MDHS	Malawi Demographic and Health Survey
MMR	Maternal Mortality Ratio
SPSS	Statistical Package for Social Sciences
VIF	Variance inflation factor
WHO	World Health Organization
